# Impacts of Intralipid on Nanodrug Abraxane Therapy and on the Innate Immune System

**DOI:** 10.1038/s41598-020-59813-7

**Published:** 2020-02-18

**Authors:** Yen-Ju Chen, Chin-Yi Tsai, Ying-Min Cheng, Su-Wen Nieh, Teng-Kuang Yeh, Ching- Ping Chen, Min-Hsien Wang, Ling-Hui Chou, Tai-Yu Chiu, Li Liu, Chien Ho, Chiung- Tong Chen, Tsang-Wu Liu

**Affiliations:** 1 National Institute of Cancer Research, National Health Research Institutes, Miaoli, County Taiwan; 2 Institute of Biotechnology and Pharmaceutical Research, National Health Research Institutes, Miaoli, County Taiwan; 3 National Institute of Neurological Disorders and Stroke, National Institutes of Health, Bethesda, MD USA; 4 Department of Biological Sciences, Carnegie Mellon University, Pittsburgh, PA USA

**Keywords:** Drug delivery, Immunology

## Abstract

A major obstacle to nanodrugs-mediated cancer therapy is their rapid uptake by the reticuloendothelial system that decreases the systemic exposure of the nanodrugs to tumors and also increases toxicities. Intralipid has been shown to reduce nano-oxaliplatin-mediated toxicity while improving bioavailability. Here, we have found that Intralipid reduces the cytotoxicity of paclitaxel for human monocytic cells, but not for breast, lung, or pancreatic cancer cells. Intralipid also promotes the polarization of macrophages to the anti-cancer M1-like phenotype. Using a xenograft breast cancer mouse model, we have found that Intralipid pre-treatment significantly increases the amount of paclitaxel reaching the tumor and promotes tumor apoptosis. The combination of Intralipid with half the standard clinical dose of Abraxane reduces the tumor growth rate as effectively as the standard clinical dose. Our findings suggest that pre-treatment of Intralipid has the potential to be a powerful agent to enhance the tumor cytotoxic effects of Abraxane and to reduce its off-target toxicities.

## Introduction

Cancer incidence increases globally along with the aging of the population and the industrialization of human society. Globally, there were an estimated 18.1 million new cancer cases and 9.6 million cancer deaths in 2018^1^. The burden of cancer care has exerted a detrimental effect on economic development that will only increase. The global economic cost of cancer in 2010 was estimated to be US$ 1.16 trillion^2^. According to the recent statistical report from the Taiwan National Health Insurance Administration, cancer therapy accounted for about 40% of the expenditure for all types of major illness, while about 38% of the cost for cancer therapy was attributed to anti-cancer drugs. In addition, the anti-cancer drug cost has gradually increased over the years with a 5.8% average growth rate during 2012~2016^3^. The reason for the rapid increase of anti-cancer drug expenditure is not only the growing number of cancer patients but also the high cost of emerging novel anti-cancer therapies, such as targeted small molecules and biological agents. Improving the efficacy of cancer drugs is a solution for reducing drug costs.

Nanomedicine is an emerging area of novel technology in cancer therapy. Nanodrugs have features of enhanced permeability and retention (EPR) in solid tumors and targeted delivery to specific tissues that can provide maximized pharmacological effects in malignant tissues and minimize the side effects in healthy organs^4,5^. However, only a small fraction of injected nanomedicines (less than 1%) is present in the tumor^6^, and the majority of nanomedicines end up in the reticuloendothelial system (RES), in particular taken up by Kupffer cells in the liver^7,8^. Size changes, surface modifications with charges or specific proteins, or combination therapy with appropriate adjuvants are reported to improve the delivery rate of nanodrugs to the tumor tissues^4,9^.

Recently, a new methodology shows great potential for improving the delivery and reducing the toxic side effects of anti-cancer nanomedicines^10–13^. Intralipid is a safe lipid emulsion used as a nutritional supplement for human patients. Pre-treatment with Intralipid reduces the toxic side effects of an experimental platinum (Pt)-containing anti-cancer nanodrug in the liver, spleen, and kidney, and improves the bioavailability of the drug in the blood^10,14^. In an HT- 29 human colon cancer xenograft mouse model, Intralipid treatment reduces the damage in bone marrow caused by Pt-nanodrugs^11^. Three FDA approved nanodrugs, Abraxane, Marqibo, and Onivyde, have also been tested with Intralipid treatment in rats. Reduced off-target toxic side effects are observed in RES organs^11^. Pre-treatment with Intralipid provides a potential “stealth” strategy to protect RES organs from nanodrug-mediated damage^11–13^.

Intralipid also shows the potential for modulating innate immune responses, which are critical for cancer immunity. It has been shown that two types of macrophages play different roles in cancer development. The classically activated M1 macrophages provide anti-tumor activities by releasing inflammatory cytokines. By contrast, the anti-inflammatory M2 macrophages play roles in promoting angiogenesis and metastasis, and suppress the immune response^15–18^. It has been reported that lipid emulsions with various compositions of fatty acids modulate macrophage polarization and cytokine production^19–21^. Our results suggest that Intralipid does not affect endocytosis, but promotes macrophage polarization into the M1-like phenotype. Thus, pretreatment with Intralipid appears to be a promising method to increase the efficacy of nanodrugs and decrease their toxicity. Here, we describe studies of the impact of Intralipid on cancer treatment in combination with the nanodrug Abraxane.

Abraxane is an albumin-bound paclitaxel nanoparticle. It binds to microtubules and interferes in the mitotic process, thus suppressing tumor growth efficiently^22^. Abraxane has FDA approval^23^ and is widely used in treating metastatic breast cancer as well as lung cancer and pancreatic cancer^23^. Sales of Abraxane alone for these several oncology indications were estimated to be $967 million, making it one of the best-selling nanodrugs in the world^24^. Breast cancer is the most commonly occurring cancer in women and the second most common cancer worldwide. The incidence rates for breast cancer far exceed those for other cancers in women. There were 2.1 million newly diagnosed breast cancer cases worldwide in 2018, accounting for almost 1 in 4 cancer cases among women^1^. According to the latest Taiwan cancer registry annual report, up to 12,672 women in Taiwan suffered from breast cancer in 2016^25^. The incidence and mortality rates of breast cancer rank as the first and the third of female cancers in Taiwan, respectively^25^. Patients with systemic metastatic breast cancers are associated with a poor prognosis, with five-year overall survival rate at about 18.5% and 22% in Taiwan and in the United States, respectively^26–28^. Increasing the delivery of Abraxane in patients with metastatic breast cancer could improve their survival rates.

In the present study, a murine mammary carcinoma model is used in Balb/c mice to observe if Intralipid enhances the ability of Abraxane to inhibit tumor growth. It is noteworthy that our results indicate that the efficacy of a combination treatment of low-dose Abraxane (half the clinical standard dose) and Intralipid is similar to that of the standard full dose treatment of Abraxane alone. These results indicate that the combination treatment of Intralipid with Abraxane enhances the inhibition of tumor growth of the drug in our mouse breast cancer model. Therefore, the combination of anti-cancer nanodrugs with Intralipid may serve as a novel strategy to improve therapeutic efficacy in treating cancers in clinical practice.

## Results

### Intralipid protects the viability of human monocytic cells, but not breast, lung, or pancreatic cancer cells in the presence of paclitaxel

**Figure 1:**
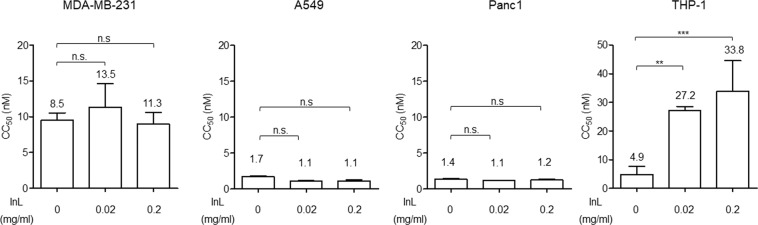
Intralipid protects the immune cells from paclitaxel *in-vitro*, but no effect is observed in human cancer cell lines. Indicated cells treated with various concentrations of Intralipid and three-fold serial diluted paclitaxel were subjected to cell-proliferation assays using a CCK-8 kit. The values summarized in the chart denote the CC_50_ values as mean ± SD from four independent experiments. Two-way ANOVA was used to evaluate the significance of the difference between the indicated data sets. ***p < 0.001; **p < 0.01; ns, not significant.

In these *in-vitro* experiments, paclitaxel, not bound to albumin, was used. In order to examine if Intralipid can enhance the cytotoxicity of paclitaxel for tumor cells, we have performed *in-vitro* cell-proliferation assays on human breast cancer (MDA-MB-231), non-small cell lung cancer (A549), and pancreatic cancer (Panc1) cell lines. In addition, the effects on the human monocytic cell line, THP-1, were analyzed in parallel. Cells were co-treated with two concentrations of Intralipid and serial-diluted paclitaxel for 3 days. The cell viabilities were analyzed using a CCK-8 kit, and the results are summarized in Fig. 1. The 50% cytotoxicity concentrations (CC_50_) of paclitaxel are 8.5 ± 4.2, 1.7 ± 0.2, 1.4 ± 0.3 and 4.9 ± 2.8 nM for MDA-MB-231, A549, Panc1 and THP-1 cells without Intralipid treatment. Intralipid at 0.02 and 0.2 mg/ml does not significantly alter the CC_50_ values for paclitaxel in cancer cells, namely 13.5 ± 4.0 and 11.3 ± 3.3 nM in MDA-MB-231 cells, 1.1 ± 0.1 and 1.1 ± 0.3 nM in A549 cells, and 1.1 ± 0.1 and 1.2 ± 0.2 nM in Panc1 cells. Interestingly, the CC_50_ values for paclitaxel significantly increase about 5-fold in THP-1 cells, namely 27.2 ± 1.3 and 33.8 ± 10.7 nM in 0.02 and 0.2 mg/ml Intralipid-treated cells, respectively. These results indicate that Intralipid has the potential to specifically protect immune cells from paclitaxel, but without affecting the cytotoxicity for tumor cells.

### *In-vitro* treatment with Intralipid promotes the polarization of macrophages to the M1-like phenotype

In order to further elucidate the effects of Intralipid, THP-1 cells were differentiated into M0, M1, and M2 macrophages by chemical induction and then incubated with Intralipid for the following functional assays. First, the capacity for endocytosis in various sub-types of macrophages was analyzed. Well-differentiated macrophages were pre-treated with Intralipid for one hr and washed with PBS. FITC (fluorescein isothiocyanate)-conjugated nano-sized beads were then added for 45 mins. The cells were fixed by neutral formalin and the numbers of green fluorescence markers in cells observed by fluorescence microscopy. The endocytic abilities are not altered among these sub-types of macrophages with or without Intralipid treatment.

**Figure 2:**
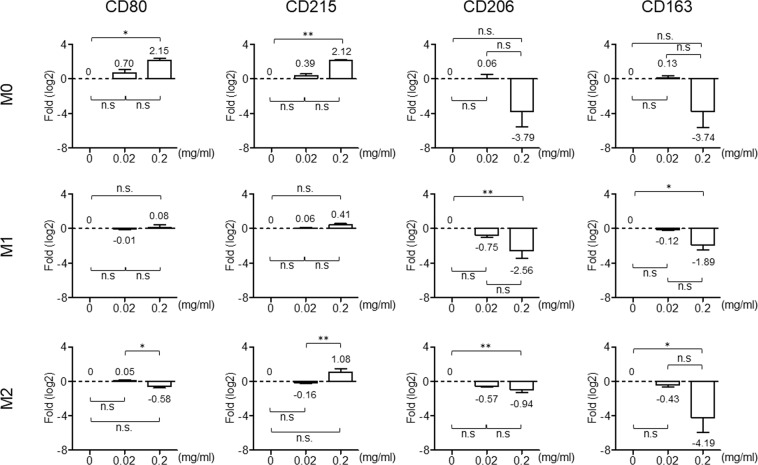
*In-vitro* treatment with Intralipid promotes the polarization of macrophages to an M1-like phenotype. After chemical induction, well-differentiated M0/M1/M2 macrophages were treated with the indicated concentrations of Intralipid (0, 0.02, 0.2 mg/ml) for 72 hrs. Total RNAs were harvested and analyzed by Q-PCR with specific primers against M1 (CD80 and CD215) and M2 (CD206 and CD163) macrophage markers. The expression levels of IPO8 served as internal controls. Data are presented as the mean ± SD of six independent experiments. Results are expressed as how many fold (log2) the expression markers increased or decreased relative to the baseline. The values summarized in the chart denote the relative expression levels in each subtype of macrophage with and without Intralipid treatment. One-way ANOVA was used to evaluate the significance of the difference between the indicated data sets. **p < 0.01; *p < 0.05; ns, not significant.

Second, we have further tested the polarization of macrophages in an Intralipid containing environment. After chemical induction, M0/M1/M2 macrophages were incubated with Intralipid for three days followed by real-time PCR analysis to determine the CD (cluster of differentiation) markers of macrophages. Expression levels of markers for each macrophage sub-type before Intralipid treatment were determined as the baseline. As previously reported, CD80 and CD215 are expressed in M1 macrophages, whereas CD206 and CD163 are present in M2 macrophages compared to the M0 subtype. As shown in Fig. 2, after three days incubation with Intralipid, in M1 macrophages, the expression levels of CD80 and CD215 are maintained, and those of the M2 markers are decreased. In M0 and M2 macrophages, the expression levels of M1 markers are elevated more than 2 fold with statistical significance, except for CD80 in M2 macrophages, where slight changes (about 1.49 fold) are observed. Moreover, CD206 and CD163 are obviously decreased in M0 and M2 macrophages with sub-type treatment.

### Treating macrophages *in vivo* with Intralipid does not affect phagocytosis, but promotes polarization into the M1-like phenotype

**Figure 3:**
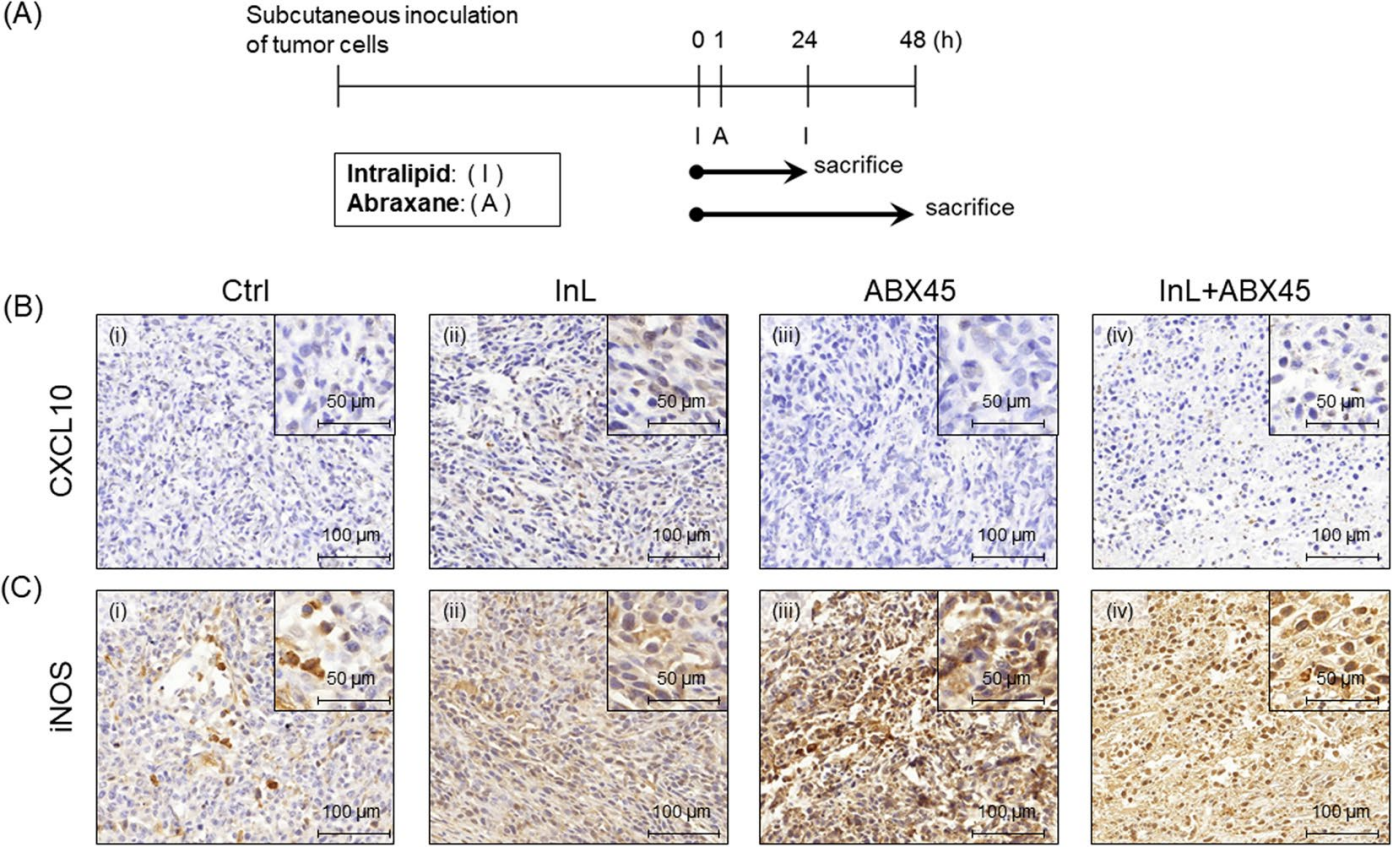
Expression levels of CXCL10 and iNOS in tumor sections indicate that *in-vivo* Intralipid does not affect phagocytosis and may promote macrophage polarization into the M1 phenotype. (**A**) Experimental Design. One dosing cycle was administered in the xenograft mouse mammary tumor model. Mouse samples were collected at the indicated time points. (**B,C**) Tumor sections of murine tumor cells with short-term Intralipid and Abraxane treatment for 48 hrs were subjected to immunohistochemistry for mouse CXCL10 (**B**) and iNOS (**C**). Original: 200x magnification; Inserts: 400x magnification.

In order to elucidate the differentiation of macrophages *in vivo* after Intralipid treatment, a xenograft mouse breast tumor model was established. Murine mammary carcinoma cells, 4T1-luc2-tdTomato, were inoculated into the left backs of Balb/c mice and allowed to grow until the tumor size reached about 200 mm^3^. These tumor-bearing mice were injected with saline alone (control), Intralipid (2 g/kg) alone, and two different dosages of Abraxane (90 or 45 mg/kg) with and without the combination therapy of 2 g/kg Intralipid. These mice were harvested at 2 time-points: 24 hrs after the first Intralipid and/or Abraxane treatment and 24 hrs after a 2^nd^ cycle of Intralipid administration (Fig. 3A). The tumor tissues were subjected to immunohistochemistry analysis for the CXCL10 chemokine and iNOS cytokine of M1 macrophages^29^. As indicated in Fig. 3B,C, fewer of the tumor tissues show positive staining of CXCL10 and iNOS in the groups treated with saline (Ctrl) or Abraxane alone (Fig. 3Bi and iii,Ci and iii). In contrast, most of the tumor tissues in groups Intralipid-treated, with or without Abraxane treatment, display enhanced positive staining of CXCL10 and iNOS (Fig. 3Bii and iv, Cii and iv). Based on these results, we postulate that incubating macrophages with Intralipid does not affect phagocytosis, but has the potential to promote macrophage polarization into the M1-like phenotype. However, more experiments are needed to further verify this issue.

### Intralipid enhances the cytotoxicity of Abraxane in tumors

**Figure 4:**
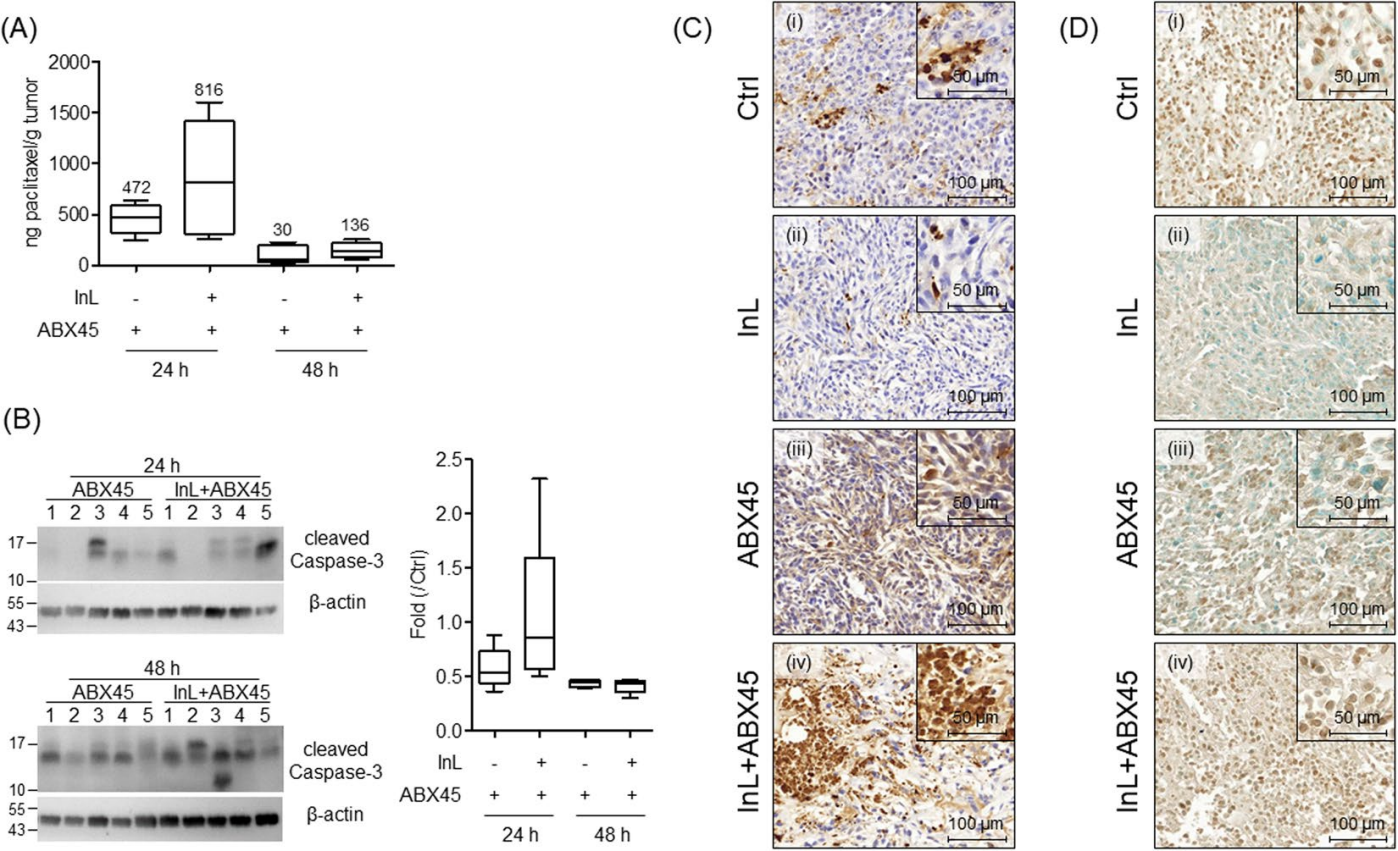
Intralipid enhances the cytotoxicity of Abraxane in tumors. (**A**) The box-and-whisker plot represents the amount of paclitaxel in tumors determined by LC/MS. The number above the box denotes the median amount of remaining paclitaxel in the tumor tissues. (**B**) Western blot analysis of the apoptosis signal, cleaved caspase-3, in the tumors at 24 and 48 hrs after Abraxane treatment. β-actin served as a loading control. The blots of caspase-3 and β-actin are cropped from different parts of the same gel. The intensity of each band of the cleaved caspase-3 was measured by ImageJ software (NIH) and normalized to its own signal of β-actin. The values summarized in the box-and-whisker plot denote the relative intensities compared with those in the control group. (**C,D**) The immunohistochemistry of cleaved caspase-3 (**C**) and the TUNEL assay (**D**) are analyzed in 48 hrs tumor sections. Original: 200x magnification; Inserts; 400x magnification.

The efficacy of the delivery of Abraxane to tumor tissue in Intralipid-treated groups was determined by using a short-term treatment with one dosing cycle in the xenograft mouse breast tumor model. Tumor tissues were analyzed for the amounts of paclitaxel present by using mass spectrometry. These results are summarized in Fig. 4A. In the groups with Abraxane treatments, a larger amount of paclitaxel (a median amount of 472 ng paclitaxel/g tumor) was detected in 24 hrs after the drug administration and declined to about 6.4% at 48 hrs (median of 30 ng paclitaxel) subsequently. In addition, we have found that pre-treatment with Intralipid significantly elevates the amount of paclitaxel in tumor tissues at 24 hrs (median of 816 ng paclitaxel), which is about 1.7-fold more than that in the group with Abraxane alone. However, at 48 hrs, no difference is observed between groups with Abraxane alone and the combination therapy (median of 136 ng paclitaxel). In groups with saline or Intralipid alone treatment, no paclitaxel was detected in the tumor tissues.

Next, we would like to elucidate if the Intralipid treatment enhances cytotoxicity in the tumors after Abraxane administration. Tumor tissues were subjected to western blot analysis for the cleaved form of caspase-3 as an indicator of apoptosis. As the data show in Fig. 4B, higher expression levels of the cleaved caspase-3 are detected in the groups with the combination therapy at 24 hrs compared to those in the Abraxane alone group. The blots of caspase-3 and β- actin are cropped from different parts of the same gel. Immunohistochemistry analysis of the cleaved caspase-3 was also performed on the tumor sections. As the data show in Fig. 4C, Abraxane alone treatment indeed induces apoptosis signals in tumor tissues. About 40~60% of the tumor tissues presented positive staining of the cleaved caspase-3 (Fig. 4Ciii). Enhanced apoptosis signals are observed in tumors with combined Intralipid and Abraxane treatments. About 70–90% of tumor sections display large amounts of apoptosis signals (Fig. 4Civ). The TUNEL assay, a sensitive method for detecting DNA fragmentation in apoptotic cells, was also performed on tumor tissues (Fig. 4D). Similar to the results in caspase-3 staining, positive signals are significantly enhanced in tumor tissues from mice with combination therapy. Based on the above results, pre-treatment with Intralipid promotes paclitaxel accumulation in the tumor tissues and induces a higher level of apoptosis of the tumor cells.

### Intralipid increases the distribution of paclitaxel in mouse RES organs but does not significantly increase apoptosis in liver tissues 48 hrs after treatment

**Figure 5:**
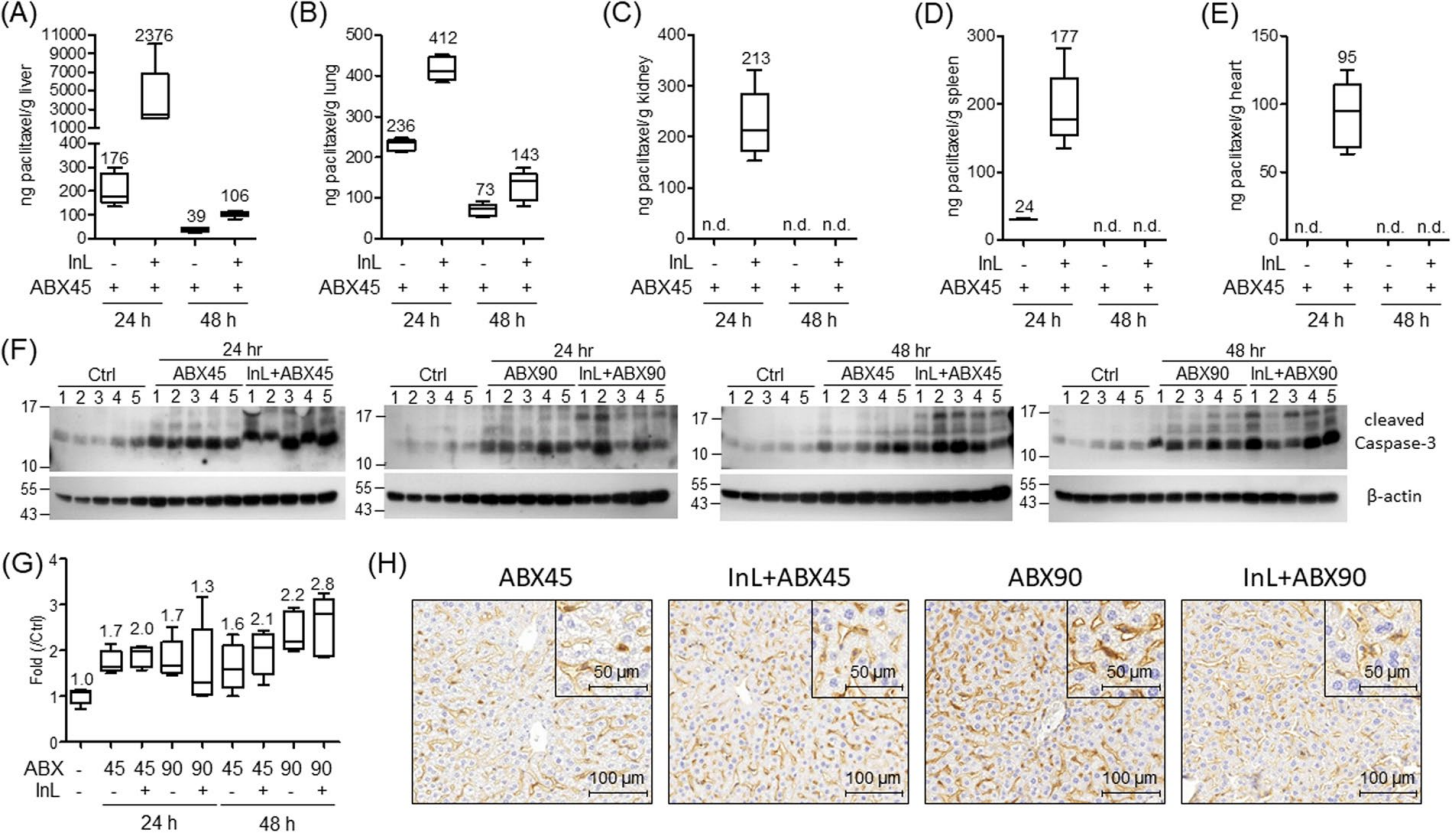
Intralipid enhances the biodistribution of paclitaxel in mice organs. (**A**–**E**) Determination by LC/MS of biodistribution of paclitaxel in mice organs at 24 or 48 hrs after Abraxane treatment. The organs include liver (**A**), lung (**B**), kidney (**C**), spleen (**D**), and heart (**E**). The number above the box denotes the median amount of remaining paclitaxel in mice organs. n.d., not determined. (**F**) Western blot analysis of the apoptosis signal of the cleaved caspase-3 in the liver at 24 and 48 hrs after treatment with 45 or 90 mg/kg Abraxane with or without Intralipid. The blots of caspase-3 and β-actin are cropped from different parts of the same gel. (**G**) The intensities of the apoptosis signals normalized with β-actin are quantified, and the values summarized in the right box-and-whisker plot, denote the relative intensities compared with those in the control group. (**H**) Immunohistochemistry of the cleaved caspase-3 in liver sections. Immunohistochemistry was performed on liver tissues in each group, and one case is shown. Original: 200x magnification; Inserts: 400 magnification.

One of the challenges of nanomedicines is their uptake by RES organs, which causes damage to surrounding normal tissues. Given the above results, we were curious if Intralipid enhances cytotoxicity in normal tissues. Therefore, liver, lung, kidney, spleen, heart, and blood of the xenograft tumor mice with short-term drug treatments were harvested. First, the amounts of remaining paclitaxel in the tissues at 24 or 48 hrs after the Abraxane treatment were measured. These results are summarized in Fig. 5A–E. At 24 hrs, larger amounts of paclitaxel are observed in liver, lung, kidney, spleen, and heart in the groups with the combination therapy compared to those with Abraxane alone treatment, especially in the liver, where about a 10-fold increase is detected. 24 hrs later, some remaining paclitaxel was observed in livers and lungs, with the levels decreased to about 100 ng paclitaxel per gram of tissue. No paclitaxel was detected in the blood samples from these mice. Further, the degree of the liver damage at 24 and 48 hrs was examined, and the cleavage form of caspase-3 was used as an indicator for apoptosis. As shown in Fig. 5F, the blots of caspase-3 and β-actin are cropped from different parts of the same gel. As the results show in Fig. 5F,G, the median damage levels of livers in both 45 and 90 mg/kg Abraxane treated mice are 1.7 fold compared to that in control mice at 24 hrs. The damage level is not altered in 45 mg/kg Abraxane-treated mice (1.6 fold) but slightly increased to 2.2 fold in 90 mg/kg Abraxane-treated mice at 48 hrs. The combination therapy with Intralipid does not significantly enhance apoptosis in liver tissues. The median damage strengths are about 1.3 to 1.7 fold at 24 hrs and 2.1 to 2.8 fold at 48 hrs compared to that in control mice. In parallel, the immunohistochemistry analysis of the cleaved caspase-3 was performed in liver tissues (Fig. 5H), and the results are similar to those in the western blots. Intralipid treatment somewhat enhances the cytotoxic effects of Abraxane in liver tissues. It is noteworthy that, in comparing the groups with 90 mg/kg Abraxane treatment, lower apoptosis signals are detected in the groups with the combination therapy of Intralipid and 45 mg/kg Abraxane. These results suggest that since combination therapy with Intralipid decreases the dose of Abraxane needed in treating cancers, it therefore also protects the RES organs from some of the chemical damage caused by the drug.

### Intralipid enhances the inhibition of tumor growth by Abraxane in a xenograft murine breast cancer model

**Figure 6:**
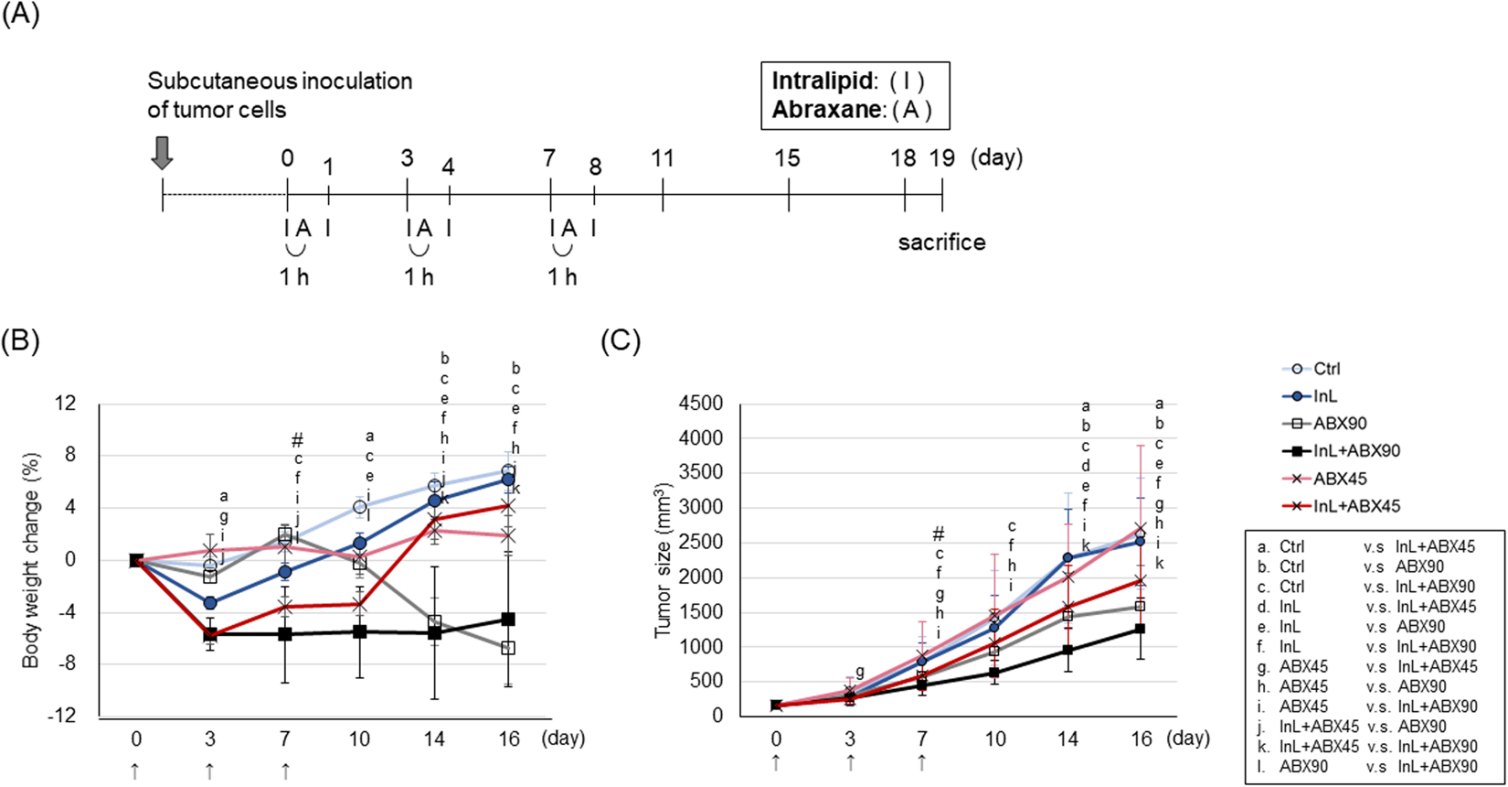
Intralipid improves the efficacy of Abraxane in treating breast cancer. (**A**) Experimental Design. Sixty Balb/c mice (N = 10 for each group), inoculated subcutaneously with 4T1-luc2-tdTomato cells, were divided into 6 groups. When the tumors had grown to a size of approximately 200–300 mm^3^, the study was initiated. One dosing cycle included two doses of Intralipid and one dose of Abraxane. The full dosing schedule included three cycles. After the final dosing cycle, the mice were monitored for nine more days, followed by sacrifice. (**B**,**C**) Comparison of body weight changes (**B**) and tumor growth inhibition (**C**) in mice with indicated treatments. Statistical significance was determined by One-Way ANOVA followed by Fisher’s LSD posthoc analysis. Data are shown as mean ± SEM. Between two groups, the significant difference is set at *p* < 0.05. #, half of the mice in the group with combination therapy of Intralipid and 90 mg/kg Abraxane were dead on day 7 after the first drug administration. Ctrl, control group; InL, Intralipid; ABX45/90, Abraxane 45/90 mg/kg.

In order to elucidate the efficacy of the combination therapy of Intralipid and Abraxane, a mouse breast tumor model with long-term treatment was established. Figure 6A shows the regimen of three dosing cycles administered, one dosing cycle consisting of a tail-vein intravenous (i.v.) injection of Intralipid before and after Abraxane i.v. administration. Tumor sizes and mouse body weights were observed twice a week until the end of the experiments. The results of the change in body weight are shown in Fig. 6B. In the control mice, the body weights gradually increase every 3-to-4 days at a rate of 2%. Treatment with Intralipid slightly interferes with the body weight increase at the beginning of the experiment (~3%, at day 3), but shows no significant difference compared to the control group, and after day 7 the body weight gain is similar to that of the control group. The body weights in the mice with 45 mg/kg Abraxane alone (half the clinical equivalent dose) stay at a stable level through the whole period of the experiment. There is some body weight lost in the combination treatment group with Intralipid and 45 mg/kg Abraxane during the dosing period. After stopping the injections, the body weights gain back to the control level and increase stably until the end of the experiment. Compared to the control group, more body weight is lost in the mice receiving the clinical equivalent dose of Abraxane (90 mg/kg) administrated with or without Intralipid (5~7%). GOT/GPT and BUN/creatinine (CRE) were measured to monitor the functions of the liver and the kidney, respectively. Neither Abraxane alone nor combination treatment with Intralipid and Abraxane significantly affect the blood levels of these indicators (Fig. S1A). The protein levels of the cleavage form of caspase 3 in mice liver were also evaluated. They are elevated in mice liver treated with the higher clinical equivalent of Abraxane compared to those treated with the half-clinical equivalent dose of Abraxane. Combination treatment of Intralipid and the half-clinical equivalent dose of Abraxane does not enhance the signals (Fig. S1B). Among these studies, five mice out of ten were dead on the 7^th^ day in the combination treatment of Intralipid and high dose Abraxane (90 mg/kg).

The alterations of tumor volumes are shown in Fig. 6C. On 16 days after the first drug treatment, there are no significant differences in tumor size between treatments with saline (2,632 ± 801 mm^3^), Intralipid (2,514 ± 627 mm^3^), and the low dose of Abraxane alone (2,714 ± 1183 mm^3^), whereas administration of the high dose of Abraxane significantly diminishes the tumor volumes (1,579 ± 605 mm^3^) by day 16. In addition, the combination with Intralipid lowers the tumor volumes in both low (1,952 ± 636 mm^3^) and high (1,263 ± 443 mm^3^) doses of Abraxane for an average 20.0% and 28.1% tumor size reduction, respectively. Notably, the repression of the tumor growth curve of the mice receiving the combination therapy of Intralipid and low dose Abraxane is close to that of the high dose of the Abraxane alone treated group. These results indicate that the combination treatment of Intralipid with Abraxane enhances the inhibition of tumor growth by the drug in our mouse breast cancer model.

## Discussion

Nanomedicines have unique properties that overcome some obstacles of conventional chemotherapy in cancer patients^30^. The nanoscale size and large ratio of volume to surface area enhance the ability of intracellular uptake and make the drug more chemically reactive^31,32^. However, the nanoparticles are easily taken up by the RES organs^31^ and therefore have toxic effects. Several strategies have been developed to resolve the dilemma, which include surface modification with charges or hydrophilic moieties to minimize recognition by phagocytic cells^33^. Lipid-based modifications, combined with targeting the tumor microenvironment, elevate the efficacy of nanomedicines in treating cancers^34,35^. Co-delivery of appropriate materials with nanomedicines is another approach. For example, the nano-sized inhibitors of hepatic cytochrome P450 3A4 (CYP3A4) serve as a priming agent to block hepatic metabolism specifically following treatment with nanomedicine, which increases the drug bioavailability and prolongs the survival of the xenograft mice^36,37^. Here, we have provided a novel strategy by using an FDA-approved nutritional supplement, Intralipid, to enhance the drug bioavailability (Fig. 4) and to attenuate tumor growth efficiently (Fig. 6). Thus, a lower dose of Abraxane can be used to reach similar anti-cancer effects, with less damage to the RES organs. Intralipid is composed of soybean oil, egg yolk phospholipid, and glycerin, which provide an important source of energy and supply the essential fatty acids^38^. In our studies, the cytotoxicity is not increased in normal tissues with a combination therapy of Intralipid and Abraxane compared to that in the group treated with Abraxane alone (Fig. 5). In addition, Intralipid does not affect the viability of immortalized human oral fibroblasts in *in-vitro* assays, even in high concentrations. Our findings suggest that pre-treatment with Intralipid could play an important role in safely enhancing nanomedicine-mediated cancer treatment.

The major component of Intralipid is neutral triglycerides of predominantly unsaturated fatty acids. The metabolic rates of neutral triglycerides are about 60 mins in adult rats and 80–100 mins in adult human after intravenous injection^39–41^. In a rat system for analyzing the toxic side effects^11^, both regimens with two or three doses of Intralipid plus one dose of nanodrugs obviously attenuate the cytotoxicity in the RES organs. As shown in both the pre-clinical mouse model and human trials, Abraxane is rapidly distributed into various tissues, reaching high drug concentrations in tissues with short duration^32,42^. Therefore, two doses of Intralipid per dosing cycle in our studies could sufficiently enhance the bio-availabilities and reduce the cytotoxicity of Abraxane. In contrast, the nano-formulations of vincristine and irinotecan, Marqibo and Onivyde, display different characteristics from Abraxane. Marqibo and Onivyde have been demonstrated to be nanoparticles with a longer circulation time and slow- release rate^43^. Because the metabolic rates of various lipids and nanomedicines are different, dosing sequences of Intralipid and nanomedicine in treating cancers of bigger animals or human should be carefully designed in order to achieve optimal efficacy.

Macrophages play important roles in maintaining physiological functions, such as engulfing and digesting pathogens, cell debris, and foreign substances, as well as secreting inflammatory mediators to activate appropriate immune responses^44^. Changes of environment, such as amounts of fatty acids, can affect the polarizations and functions of macrophages. Saturated fatty acids enhance an M1-like differentiation, whereas the M2 sub-type is induced by longer, more unsaturated fatty acids^20^. The oxidations or metabolites of fatty acids promote macrophages differentiating into the M2 subtype^45^, whereas circulating levels of free fatty acids elevate the populations of M1 subtypes^46^. Moreover, chronic treatment with palmitate decreases phagocytosis and inhibits the production of proinflammatory cytokines^47^. In the present study, the *in-vitro* macrophage differentiation model is established to observe the alternations of macrophages in an Intralipid-containing environment. As previously reported, the endocytic abilities of macrophages are not changed after Intralipid treatment^48^. M1-like populations are increased in Intralipid-treated macrophages (Figs. 2 and 3). Intralipid protects THP-1 monocytes from the cytotoxic effects of paclitaxel without compromising their efficacy for treating cancer cells (Fig. 1). Based on these results, we postulate that Intralipid could be a powerful agent with high clinical potential for nanomedicine-mediated cancer therapy.

In summary, this study of a xenograft murine tumor mouse model shows that Intralipid pre-treatment elevates the delivery rate of Abraxane into tumor cells. Combination therapy with the half-clinical dose of Abraxane and Intralipid achieves the same anti-tumor effects as those in groups with a clinical dose of Abraxane alone, with fewer side effects in RES organs. Through *in-vitro* studies, it has been shown that Intralipid treatment protects the immune cells, but does not affect the chemotherapeutic sensitivity to paclitaxel in breast cancer cells. Furthermore, Intralipid treatment promotes the trend of elevating populations of M1-like macrophages compared to the control cells. Therefore, the combination of anti-cancer nanodrugs with Intralipid may serve as a novel strategy to improve therapeutic efficacy in treating cancers.

## Materials and Methods

### Cell culture

4T1-luc2-tdTomato cell line, a luciferase and red fluorescent protein (tdTomato)-expressing cell line, is a gift from Caliper Life Sciences, Inc. (CA, USA). THP-1, MDA-MB-231, and Panc1 cell lines were purchased from BCRC (Bioresource Collection and Research Center, Hsinchu, Taiwan). THP-1 cells were grown in RPMI1640 medium supplemented with 10% fetal bovine serum (FBS). 4T1-luc2-tdTomato cells were maintained in RPMI1640 containing 10% FBS, and MDA-MB-231, A549, and Panc1 cells were maintained in DMEM containing 10% FBS.

### Breast cancer xenograft model in Balb/c mice

Four-to-five-week-old female Balb/c mice were provided by BioLASCO (BioLASCO Taiwan Co., Ltd., Taipei, Taiwan) and maintained at the Laboratory Animal Center of the National Health Research Institutes of Taiwan (NHRI). All procedures were approved by the Animal Experiments Review Committee of the National Health Research Institutes of Taiwan, (Protocol Number: 105–158-A-S03 and 107–122-A-S01). Animal care was provided in accordance with the guidance of the Association for Assessment and Accreditation of Laboratory Animal Care (AAALAC). One hundred thousand 4T1-luc2-tdTomato mouse breast tumor cells were mixed with MatriGel Matrix (Corning, NY, USA) and inoculated subcutaneously into the left back of Balb/c mice. When the tumors had grown to a size of approximately 200–300 mm^3^, the study was initiated. One dosing cycle included two doses of Intralipid (Fresenius Kabi Austria GmbH, Austria) and one dose of Abraxane (Celgene, NJ). The first dose of Intralipid was administered intravenously 1 hr before the treatment with Abraxane. The second dose of Intralipid was administered 24 hrs post-Abraxane treatment. Tumor diameters were measured with calipers and tumor volumes in mm^3^ were calculated as Volume % = (width × length^2^)/2. The growth curves of tumors were monitored twice per week until the tumor sizes achieved the maximum volume of mouse tolerance. After three dosing cycles, tumor growth rates were monitored for three more observation time points. All of the mice were sacrificed to harvest the tumors. For cytotoxicity assays, one dosing cycle was administered, half of the mice in each group were sacrificed at 24 hrs after the first dose of Abraxane, and the others were sacrificed at 48 hrs after Abraxane administration. After anesthetization, blood samples were harvested through cardiac puncture; subsequently, mouse organs and tumor tissues were collected.

### Western blotting

Mice tissues were extracted by RIPA buffer using MagNA Lyser Green Beads (Roche Life Science, Penzberg, Germany), subjected to SDS-PAGE separation and transferred onto methanol-activated PVDF membranes (Pall Corporation, NY, USA). The membranes were blocked for one hour at room temperature in 5% non-fat milk containing TBST and incubated with the indicated primary antibodies at 4 °C overnight. The blots were washed three times with TBST for 5 min each; subsequently, the blots were incubated with peroxidase-conjugated secondary antibody for one hour at room temperature. After appropriate washing, the blots were developed by SuperSignal^TM^ West Pico PLUS Chemiluminescent Substrate kit (Thermo Fisher Scientific, MA, USA).

### Antibodies

The following antibodies were purchased from commercial suppliers: Rabbit monoclonal antibody for cleaved caspase-3 (Cell Signaling Technology, #9664); rabbit polyclonal antibody for iNOS (Abcam, #ab15323); goat polyclonal antibody for CXCL10 (R&D system, AF-466-NA); mouse monoclonal antibody for beta-actin (Novus Biologicals, #NB600-501).

### Determination of the amounts of paclitaxel in mice tissues

The chromatographic system consisted of an Agilent 1200 series LC system and an Agilent ZORBAX Eclipse XDB-C8 column (5 µm, 3.0 × 150 mm) interfaced to an MDS Sciex API4000 tandem mass spectrometer, equipped with an ESI in the positive scanning mode at 600 °C. The MS/MS ion transitions monitored were m/z 855.2/686.2 and 357/195 for paclitaxel (analyte) and internal standard (IS), respectively. The collision energies were 27 and 35 V for the analyte and IS, respectively. A gradient HPLC method was employed for separation. Mobile phase A consisted of 10 mM ammonium acetate aqueous solution containing 0.1% formic acid and mobile phase B consisted of acetonitrile. The gradient profile was as follows (min/%B): 0.0- 1.1/20, 1.2-3.7/80, 3.8-5.0/20. The flow rate was set at 1.5 mL/min that was directed to the mass spectrometer and the remainder was split off to waste. The autosampler was programmed to inject 15 µL sample aliquots every 5 min. The retention times of paclitaxel and IS were 2.49 and 2.48 min, respectively.

### Immunohistochemistry and the TUNEL assay

Mice organs and tumor tissues were fixed in 10% formalin, processed, and embedded in paraffin. Four-micrometer tissue sections were deparaffinized, rehydrated, and retrieved by 1 × Trilogy^TM^ (Sigma-Aldrich, Denmark). Endogenous peroxidase was neutralized by 3% hydrogen peroxide (H_2_O_2_, Fluka^TM^, Germany) for 5 min. After incubation with Protein Block (Leica Biosystems Newcastle Ltd, UK), optimally diluted primary antibodies were added onto tissue sections and incubated overnight at 4 °C, followed by incubation with Post Primary Block and NovoLink Polymer (Leica Biosystems Newcastle Ltd) at room temperature sequentially. The tissue sections were developed by DAB working solution and counterstained with G/M Hematoxylin. After staining, the slides were dehydrated in ascending grades of alcohol, cleared in xylene, and mounted with xylene-based mounting medium (Muto Pure Chemicals Co. Ltd., Japan). Paraffin embedded tumor sections were used for the TUNEL assay by means of the HRP-DAB TUNEL staining kit (Ab206386, Abcam), and the slides were counterstained by methyl blue. All of the stained slides were scanned by histologic digital scanner Pannoramic MIDI (3DHISTECH Ltd, Budapest, Hungary).

### Cytotoxicity assay

Cells were seeded in 96-well plates at a density of 10,000 cells per well for THP-1 cells and 5,000 cells per well for MDA-MB-231 cells and incubated overnight. Serially diluted paclitaxel with/without Intralipid was added by medium replacement for 3 days. In cells with Intralipid pre-treatment, the indicated concentration of Intralipid was added to the cells for 1 hr before paclitaxel (Sigma-Aldrich) treatment and followed by 2 PBS washes. CCK-8 solution (Cell Counting Kit-8, Dojindo Molecular Technologies, Inc., Japan) was used to determine the number of viable cells through measuring the absorbance at 450 nm. The 50% cytotoxic concentration (CC_50_) was calculated as the concentration that inhibits 50% of the conversion of the substrates (WST-8) to formazan, which was analyzed using the GraphPad Prism 5 software (GraphPad software, CA).

### Determination of macrophage polarization

THP-1 cells were seeded in 6-well plates at a density of 10^6^ cells per well, incubated overnight, and followed by macrophage differentiation by chemical induction. To generate M0 macrophages, 50 nM PMA (Sigma-Aldrich) was added for 48 hrs. To generate M1 macrophages, cells were treated with 50 nM PMA for 6 h, followed by incubation with 10 pg/ml LPS (Sigma-Aldrich) and 20 ng/ml IFN-γ (R&D Systems, MN, USA) for 48 h. To generate M2 macrophages, cell were treated with 50 nM PMA for 6 h, followed by incubation with 20 ng/ml IL-4 (R&D Systems) and IL-13 (R&D Systems) for 72 hrs. After macrophage differentiation, cells were washed with PBS two times, treated with Intralipid for 3 days, and then total cellular mRNA was harvested by using TRIzol^TM^ Reagent (Invitrogen^TM^). One microgram mRNA was reverse-transcribed by using a High-capacity cDNA reverse transcription kit (Applied Biosystems, CA, USA) in a 20-μl reaction mixture. Appropriate amounts of the resulting cDNAs were mixed with 0.2 μM primers and Power SYBR Green Master Mix (Applied Biosystems, CA), and subjected to PCR amplification using the StepOnePlus^TM^ Real-Time PCR system (Applied Biosystems). Specific primers for human CD80 (5′- GAGGCAGGGAACATCACCAT-3′ and 5′-TCACGTGGATAACACCTGAACA-3′), CD215 (5′-TCCAGGGAGCGGTACATTTG-3′ and 5′-TTTGAGACTGGGGGTTGTCC-3′), CD206 (5′-CGAGGAAGAGGTTCGGTTCACC-3′ and 5′-GCAATCCCGGTTCTCATGGC-3′), and CD163 (5′-AAAAGAATCCCGCATTTG G-3′ and 5′-GGCTTCACTGGTCAGTCTCAG-3′) were used in the present study to determine the polarization of macrophages. The expression levels of human IPO8 (5′-TGTGTAG GAAGGTACTATGTGGAGA-3′ and 5′-GTACGAAGCTCACTAGTTTTGACC-3′) were used as internal controls.

### Statistical analysis

All experiments were performed on at least three separate occasions and the animal number in each treatment group was n ≥ 5. The quantitative data is expressed as mean ± SEM. Statistical significance was determined by One-Way ANOVA followed by Fisher’s least significant difference (LSD) posthoc analysis. SPSS version 13.0 (SPSS Inc., Chicago, IL) was used for analysis. Statistical significance was set at *p* < 0.05.

## Supplementary information

Supplementary Information.
