# Supplementary material for: Impacts of Intralipid on Nanodrug Abraxane Therapy and on the Innate Immune System
Source: Sci Rep. 2020 Feb 18;10:2838. doi: 10.1038/s41598-020-59813-7 (PMC7028991; doi:10.1038/s41598-020-59813-7)
Supplement: Supplementary file 1 — Supplementary Information. [file 41598_2020_59813_MOESM1_ESM.docx]

**Supplementary Information**

## Impacts of Intralipid on Nanodrug Abraxane Therapy and on the Innate Immune System

Yen-Ju Chen^1^*, Chin-Yi Tsai^2^*, Ying-Min Cheng^1^, Su-Wen Nieh^1^, Teng-Kuang Yeh^2^, Ching- Ping Chen^2^, Min-Hsien Wang^2^, Ling-Hui Chou^2^, Tai-Yu Chiu^2^, Li Liu^3^, Chien Ho^4†^, Chiung- Tong Chen^2†^, and Tsang-Wu Liu^1†^

^1^ National Institute of Cancer Research, National Health Research Institutes, Miaoli County, Taiwan

^2^ Institute of Biotechnology and Pharmaceutical Research, National Health Research Institutes, Miaoli County, Taiwan

^3^ National Institute of Neurological Disorders and Stroke, National Institutes of Health, Bethesda, MD, USA

^4^ Department of Biological Sciences, Carnegie Mellon University, Pittsburgh, PA, USA

* The authors contributed equally to this work.

^†^ Correspondence authors:

Chien Ho: Carnegie Mellon University, 4400 Fifth Ave Pittsburgh, PA 15213, USA., Telephone: 1-412-268-3395, E-mail: chienho@andrew.cmu.edu; Chiung-Tong Chen: No. 35, Keyan Road, Zhunan Town, Miaoli County 35053, Taiwan, Telephone: 886-37-246-166 ext. 35700. E-mail: ctchen@nhri.edu.tw; Tsang-Wu Liu: No. 35, Keyan Road, Zhunan Town, Miaoli County 35053, Taiwan. Telephone: 886-37-246-166 ext. 35120, E-mail: [walter@nhri.edu.tw.](mailto:walter@nhri.edu.tw)

**Supplementary Figure Captions**

**Fig. S1.** (A) Blood chemistry tests of GOT, GPT, BUN, and CRE were performed to evaluate the functions of liver and kidney in mice with indicated treatments and represented as box-and-whisker plots. The number above the box denotes the median value of indicated tests in each group of mice. (B) Western blot analysis of the cleaved caspase-3 in the liver at the experimental end point. β-actin served as a loading control. The blots of caspase-3 and β-actin are cropped from different parts of the same gel.


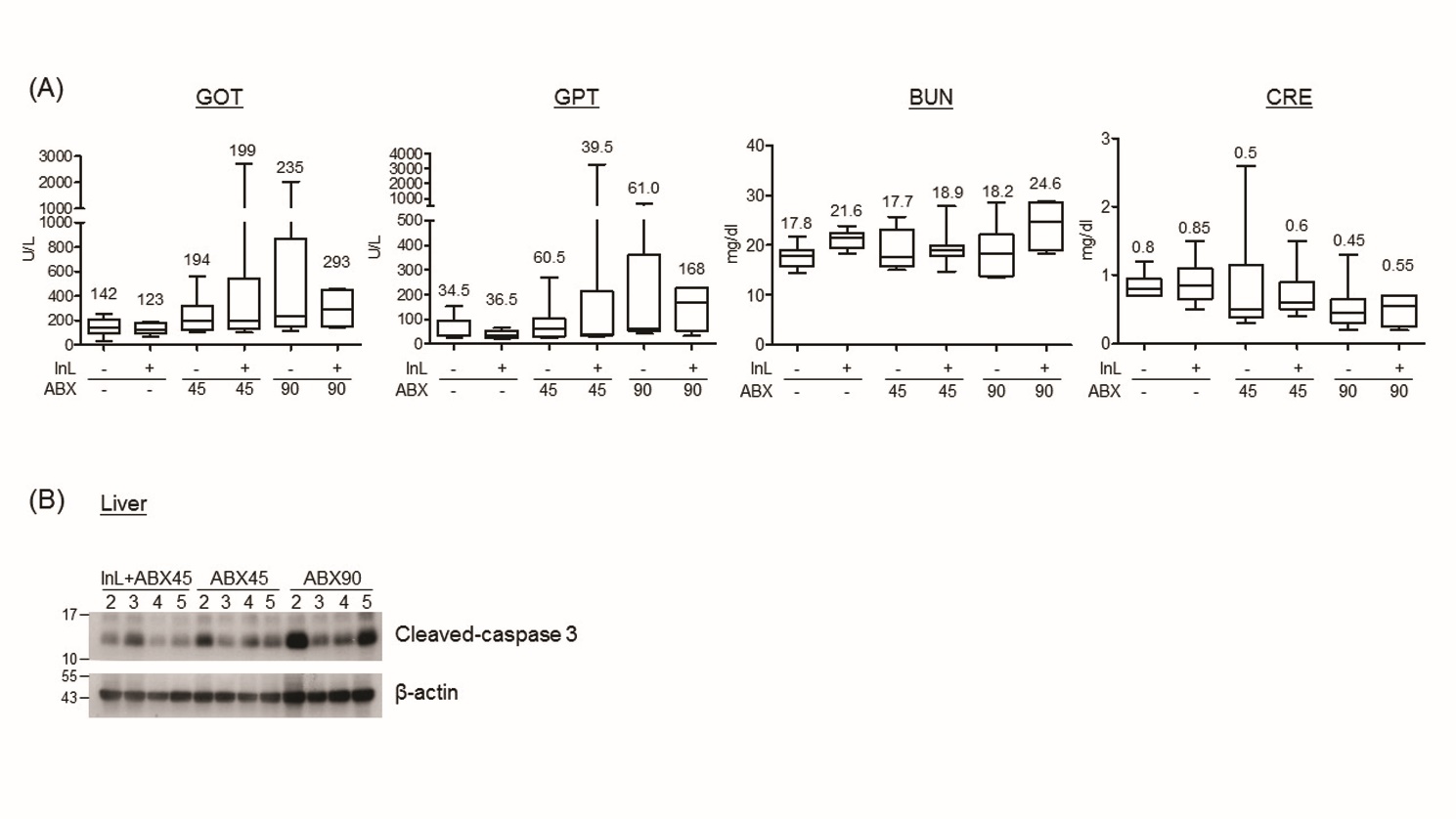


**Fig. S1**
